# Case 1 / 2019 - Natural Evolution of Double Outlet Right Ventricle with Noncommitted Ventricular Septal Defect and Pulmonary Stenosis in a 28-Year-Old Asymptomatic Woman

**DOI:** 10.5935/abc.20180260

**Published:** 2019-01

**Authors:** Edmar Atik, Alessandra Costa Barreto, Maria Angélica Binotto

**Affiliations:** Instituto do Coração do Hospital das Clínicas da Faculdade de Medicina da Universidade de São Paulo, São Paulo, SP - Brazil

**Keywords:** Double outlet Right Ventricle, Heart Septal Defects, Ventricular, Pulmonary Valve Stenosis, Clinical Evolution, Adult

## Clinical data

The patient evolved without symptoms from birth, when the diagnosis of heart disease was made, as evidenced by heart murmur. Two years ago, supraventricular extrasystoles caused the use of atenolol, propafenone, and magnesium, without improvement. Infective endocarditis was effectively treated 10 years ago. The patient uses levothyroxine 50 mcg for hypothyroidism.

Physical examination: good overall status, eupneic, acyanotic, normal pulses in the four limbs. Weight: 60 Kg, Height 160 cm, right upper extremity blood pressure: 120 x 70 mmHg, HR: 60 bpm.

Precordium: non-palpable apex beat, without systolic impulses. Hyperphonetic heart sounds, intense systolic murmur, with a thrill in the upper left sternal border, 4/6 +. Non-palpable liver and clean lungs.

### Complementary examinations

**Figure 1:**
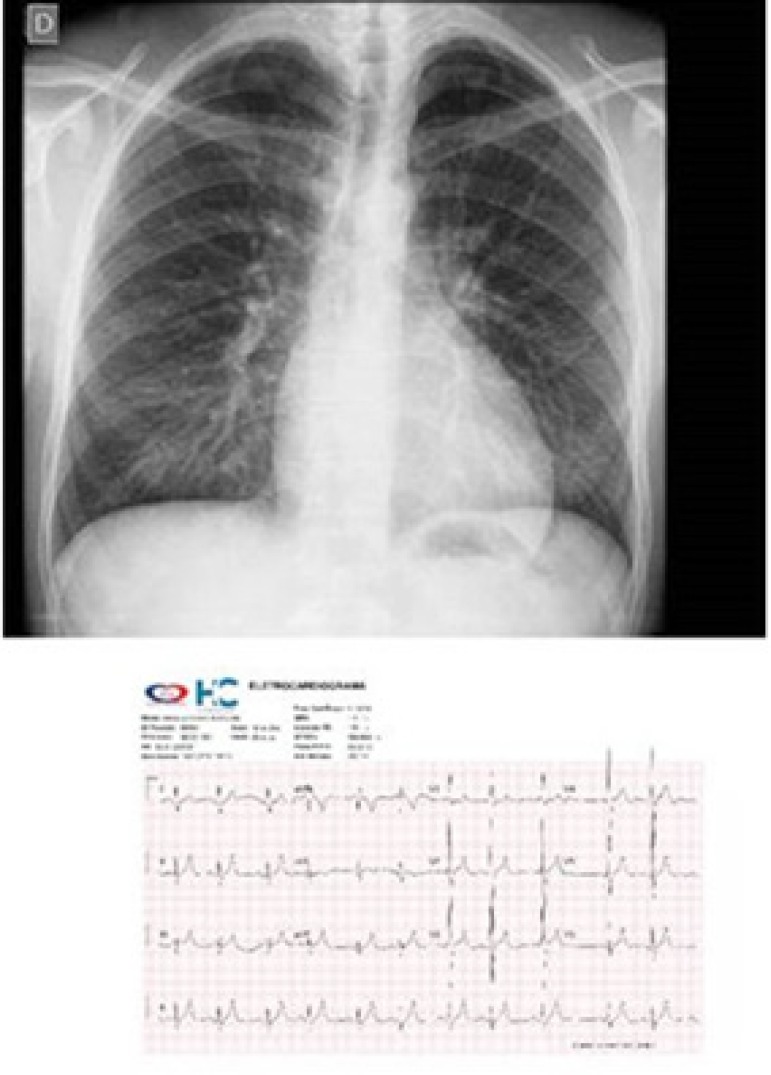
Chest X-ray showing the cardiac area within normal limits, with an elongated and rounded ventricular arch, normal pulmonary vascular network, and electrocardiogram showing signs of right ventricular overload.

**Electrocardiogram:** Sinus rhythm, right bundle-branch conduction disorder, with a wide QRS of 129 ms (AQRS = + 60º), right ventricular overload with Rs complex in V1, presence of left potentials with qRs complex in V6, positive T-wave in V1 (AT = + 60º), normal P wave (AP = + 60º) (Figure 1).

**Chest X-ray:** Slightly enlarged cardiac area, with elongated and rounded left ventricular arch (WC = 0.50). Normal pulmonary vascular network (Figure 1).

**Figure 2:**
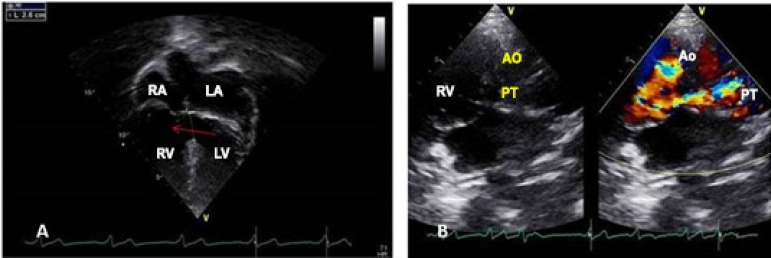
Echocardiogram shows in the 4-chamber view the large ventricular septal defect (arrow) in the inflow tract and in the subcostal view, the two large vessels emerging from the right ventricle, with the aorta to the right of the pulmonary artery. RA: right atrium; LA: left atrium; RV: right ventricle; LV: left ventricle; Ao: aorta; PT: pulmonary trunk.

**Echocardiogram**: Normal atrioventricular connection and double outlet right ventricle (DORV) with anterior aorta on the right. The inferior vena cava was dilated with 21 mm and with spontaneous contrast. The ventricular septal defect (VSD) of the inflow tract with an extension to the outflow tract was large and unrelated, measuring 23 mm, with bidirectional flow, with preferential left-to-right shunting and without restriction, with an interventricular pressure gradient of 12 mmHg. There was another discrete apical VSD. The atria were moderately enlarged (LA = 51 mm). The right ventricle was hypertrophic and dilated with preserved systolic function, infundibular and pulmonary valve stenosis in the outflow tract with a systolic gradient of 90 mmHg. The left ventricle (LV) was hypertrophic and dilated (67 mm), with normal function. The aorta measured 35 mm and the pulmonary arteries measured 31 mm to the right and 29 mm to the left (Figure 2).

**Magnetic nuclear resonance:** The diagnosis was confirmed with similar measurements: the left atrium and the two ventricular cavities were enlarged. Thus, RVEDV = 134 ml/m^2^ and RV function = 48%. LVEDV = 180 ml/m^2^ with LV function = 68%. There was late enhancement in the lower junctional region. The pulmonary artery was posterior and located to the right, whereas the aorta was anterior and located to the left.

**Holter:** Supraventricular extrasystoles (3% of the total) and no supraventricular or ventricular tachycardias.

**Ergospirometry:** Maximal oxygen consumption of 24.4 mL/kg/min.

**Clinical diagnosis:** Double Outlet Right Ventricle with anterior aorta to the right, with large unrelated inflow tract VSD and pulmonary stenosis, undergoing natural evolution in adulthood.

**Clinical reasoning:** There were clinical elements suggesting a diagnosis of congenital heart disease, with arterial malposition considering the hyperphonetic heart sounds and pulmonary stenosis in the presence of intense systolic murmur in the pulmonary area that irradiated to the entire left sternal border. The RV overload on the electrocardiogram with clear LV potentials denotes the presence of two well-formed ventricles and, hence, the presence of associated VSD is invoked. One defect offsets the other in such a way that the patient remained acyanotic, with preferential left-to-right shunting and no symptoms. This overall picture could be found in the presence of transposition of the great arteries and also in the double right ventricular outflow tract and in the tetralogy of Fallot, given the observed long-term evolution. These supposed clinical diagnoses were then well established by echocardiography and nuclear magnetic resonance.

**Differential diagnosis:** Other cardiopathies that accompany VSD and PS show other elements that differentiate them in the usual complementary examinations, such as the double LV or RV inflow tract, atrioventricular valve atresia, corrected transposition of the great arteries, and in other rarer ones.

**Conduct:** In view of the balance of the pulmonary and systemic flows over time, with the absence of signs of hypoxemia and/or heart failure and in the presence of good physical tolerance, the expectant clinical management was considered.

**Comments:** The natural evolution of this patient into adulthood emphasizes unfavorable elements, although she has been shown to be in good clinical and hemodynamic conditions. They are the acquired characteristics that interfere in the evolution over the elapsed time. In this case, they are represented by enlarged heart cavities, caused by pulmonary hyperflow at some time, and by the progression of pulmonary stenosis, with hypertrophy and even confirmed myocardial fibrosis. Despite the maintenance of good ventricular function, this patient will probably experience more arrhythmias, diastolic heart failure, progressive hypoxemia, infective endocarditis, which are the reasons for the lack of clinical control caused by the disease evolution.

On the other hand, little can be offered at this moment, from the surgical point of view, since the technique considered as adequate would be the Fontan procedure, contraindicated by the absence of hypoxia. The corrective technique would be very difficult due to the presence of the unrelated VSD and anterior aorta. Therefore, a question is raised, whether in similar cases in childhood, it would not be more convenient to attempt the correction in this age group, even with greater surgical risk. This technique, created by Barbero-Marcial et al.,^1^ directs the LV to the aorta, with ensuing pulmonary stenosis relief, and it has been applied with relative success, considering the 5-year survival rate of 87.5%.^2^
